# Supplementary material for: Favorable prognosis of patients who received adjuvant androgen deprivation therapy after radiotherapy achieving undetectable levels of prostate-specific antigen in high- or very high-risk prostate cancer
Source: PLoS One. 2021 Mar 12;16(3):e0248461. doi: 10.1371/journal.pone.0248461 (PMC7954315; doi:10.1371/journal.pone.0248461)
Supplement: Attachment — Submitted filename: Response to Reviewers.docx [file pone.0248461.s002.docx]

Reviewer #1:

(No Response)

Reviewer #3:

The major concerns is that this study did not provide novel information regarding the treatment of prostate cancer.

Reviewer #5:

1. In your table1, the percentage of 5-yr CFFS, 5-yr DMFS, 5-yr CSS in no A-ADT group is 94.1%, 94.1%,89%. Why the percentage of 5-yr CSS is lower than 5-yr CFFS, 5-yr DMFS. Does it mean that some patients died due to prostate cancer without disease progression or metastasis? You may give the numerator and denominator in each column.

**Author response:** We calculated the CSS with the information of cause of death from Department of Cancer Statistics at our institute and also from Ministry of Health in our country. However, according to your comments, we found out that some information of cause of death were wrongly gathered as ICD of prostate cancer (C61), actually even without prostate cancer progression. So we reviewed the hospital records of dead patients again and found their last follow-up date at hospital very close to date of death. We confirmed no evidence of prostate cancer progression in 5 out of 6 patients, who were initially regarded as an event of CSS. We revised the 5 cases of death as a censored at date of death without prostate cancer progression and accordingly recalculated CSS. We revised the CSS in manuscript and Table 2 and 3. The significance of A-ADT was lost in view of CSS in uni- and multivariate analysis.

In multiple variable analysis, the HR is 0.111 in ADT group compared with non- ADT group, this data is strange.
**Author response**: It means that the HR for CSS in A-ADT patients was significantly lower than those of no A-ADT by 0.111 times at initial manuscript. However, after we recalculated CSS with updated information of cause of death, the significance of A-ADT was lost in view of CSS in uni- and multivariate analysis.

2. The androgen duration in your cohort is 12-96 months. It means some patients were still under androgen therapy during 5 yrs followed-up. Hence it is not surprisingly that these patient were not experienced biochemical failure. Hence more important is DMFS, CSS in your cohort.
**Author response**: In multivariate analysis, we found that patients with A-ADT for at least 12 months survived longer significantly in view of BCFFS or OS. However, after we recalculated CSS with updated information of cause of death, the significance of A-ADT was lost in view of CSS in uni- and multivariate analysis.

3. Besides, which is more important, A-ADT, or androgen duration (N-ADT+A-ADT)?
If the zero time is date of androgen usage, were there any difference in your results?

**Author response:** N-ADT (for ≥2months prior to RT) was performed in 78 (39.6%) patients but we didn’t find any significant difference between N-ADT and no N-ADT groups in all treatment outcomes in this study. Therefore, we tried to figure out the effect of adding A-ADT with the day after the completion of RT as of zero time.

4. Since radiation therapy with 1.5-3yrs hormone therapy is the recommendation therapy in NCCN guideline. Hence, less than 1.5 yrs hormone therapy is inadequate therapy. You may exclude these patients to show the neediness of longer androgen duration.

**Author response:** As you pointed out, adjuvant ADT for 1.5-3 years is currently recommended for high risk patients. Also in our study, the patients with ≥18 months developed BCF much less frequently than the patients with less than 18 months or no A-ADT (5.8% vs. 25.6%). Moreover, BCSSF was better in the group of ADT for more than 2 years than less than 2 years (Figure 3). In this study, six patients received A-ADT for 12 - 18 months and of them, one patient developed BCF at 9 months after RT even during A-ADT. There were concerns about ADT complications which could negatively affect the quality of life with longer duration of A-ADT. Some patients discussed with urologists an intermittent A-ADT or interruption of ADT after one year of A-ADT for their quality of life or due to concurrent medical illness. Some literatures showed that ADT for more than 1 year could decrease nadir PSA effectively and improve biochemical failure-free survival (Am J Clin Oncol;40(4):348-52, Radiat Oncol 2017;12(1):149). One randomized trial of dose escalation study using EBRT or brachytherapy for high risk patients, adjuvant ADT was performed for 1 year (Int J Radiat Oncol Biol Phys 2017;98(2):275-85). So, for various reasons, we used the definition of A-ADT duration as for at least 1 year to figure out the effect of A-ADT in this study.

5. Since the novelty of your study is that psa<0.001 is an important prognostic factor. You may show more analysis regarding psa<0.001.

**Author response:** The detailed analysis of nadir PSA ≤0.001 ng/mL after RT was presented in a Table 2 and 3. We think the novelty of this study is the level of nadir PSA after RT (0.001 or less ng/mL) is much lower than other studies. This is probably due to the relatively longer duration of A-ADT with a median of 36 months in the present study compared to other studies. Nadir PSA value ≤0.001 ng/mL is also a good prognostic factor in patients received radical prostatectomy in some references. To our best knowledge, this is the first study that the nadir PSA value of 0.001 ng/mL is an independent factor on treatment outcomes in patients treated with RT and ADT in high and very high risk group.

We appreciate all your comment.
