# Supplementary material for: Favorable prognosis of patients who received adjuvant androgen deprivation therapy after radiotherapy achieving undetectable levels of prostate-specific antigen in high- or very high-risk prostate cancer
Source: PLoS One. 2021 Mar 12;16(3):e0248461. doi: 10.1371/journal.pone.0248461 (PMC7954315; doi:10.1371/journal.pone.0248461)
Supplement: Attachment — Submitted filename: Response to Reviewers.docx [file pone.0248461.s003.docx]

Reviewer #5: Since long-term adjuvant hormone therapy can not prolong CSS. the finding of long-term adjuvant hormone therapy can prolong OS is a strange finding and need to be clarified.

Answer: Patients who did not receive A-ADT had medical comorbidities more frequently than patients who received A-ADT. Among 37 patients who did not receive A-ADT, all 12 dead patients did not die of prostate cancer. However, among 11 dead patients out of 160 patients who received A-ADT, two patients died of prostate cancer and nine patients died of other causes. We thought that was why the use of A-ADT did not show significant difference in CSS but showed significant difference in OS in this study. We added this description in Discussion section.

We appreciate your review comment.
